# A ZnS@N-GQD nanocomposite as a highly effective and easily retrievable catalyst for the sonosynthesis of β-amino carbonyls

**DOI:** 10.1039/d1ra02975d

**Published:** 2021-06-03

**Authors:** Javad Safaei-Ghomi, Mohammaed Abdulridha Mutashar, Zahra Saharkhan

**Affiliations:** Department of Organic Chemistry, Faculty of Chemistry, University of Kashan Kashan 51167 I. R. Iran safaei@kashanu.ac.ir +98-31-55912397 +98-31-55912385; Department of Inorganic Chemistry, Faculty of Chemistry, University of Kashan Kashan 51167 I. R. Iran

## Abstract

A three-component reaction of acetophenone, aromatic aldehydes, and aniline derivatives has been achieved in the presence of a ZnS@nitrogen graphene quantum dot (N-GQD) nanocomposite as a highly effective heterogeneous catalyst to produce β-amino carbonyls. The catalyst has been characterized by XRD, SEM, TEM, FT-IR spectroscopy, EDS, BET and TGA techniques. The feasibility of carrying out the one-pot synthesis under ultrasonic irradiation with a heterogeneous nanocatalyst could improve the reaction rates and shorten the reaction times.

## Abstract 2

A flexible and highly efficient protocol for the sonosynthesis of β-amino carbonyls using a ZnS@N-GQD nanocomposite has been developed.

## Graphical abstract

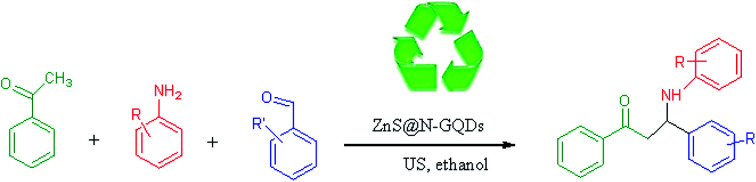

## 1. Introduction

**Scheme 1:**
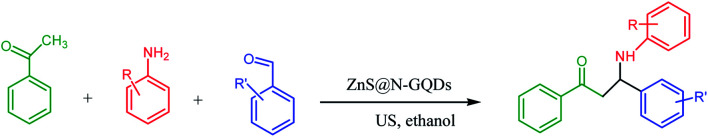
Preparation of β-amino carbonyls using the ZnS@N-GQD nanocatalyst.

β-Amino carbonyls have biological properties including anti-cancer,^1^ anti-diabetes,^2^ antibacterial, antioxidant,^3^ antifungal,^4^ anti-virus,^5,6^ and non-steroidal progesterone receptor antagonists.^7^ Therefore, searching for effective methods for the synthesis of β-amino carbonyls is an attractive challenge. β-Amino carbonyls have been regarded as notable targets in organic syntheses. A number of ways have been mentioned for the preparation of β-amino carbonyls using diverse catalysts including HClO_4_,^8^ phenyl boronic acid,^9^ Zn(OTf)_2_,^10^ indium trichloride,^11^ Zn(BF_4_)_2_,^12^ ceric ammonium nitrate,^13^ bismuth trichloride,^14^ sulfamic acid,^15^ sulfuric acid-modified polyethylene glycol,^16^ and manganese perchlorate hexahydrate.^17^ Each of these methods may have its own advantages but also suffer from apparent drawbacks such as prolonged reaction time, low yield, complicated work-up, or hazardous reaction conditions. Graphene quantum dots (GQDs) have numerous potential applications in electronics,^18^ solar cells,^19^ light emitting diodes,^20^ bioimaging,^21^ drug delivery,^22^ photocatalysts,^23^ sensors,^24^ and fuel cells.^25^ GQDs are carbon-based nanoscale particles that display excellent physical, chemical, and biological properties, which permit them to excel in a wide spectrum of applications in nanostructures.^26–31^ Sonochemistry is an essential tool in the field of synthetic organic chemistry.^32,33^ Sonochemistry is a very good method to synthesize organic compounds with high yields, short reaction time, and mild conditions.^34,35^ The effects of ultrasonic irradiation during organic reactions is due to cavitation and then the collapse of the bubbles that produce short-lived regions with high pressure and temperature.^36,37^ Herein, we report the use of the ZnS@N-GQD nanocomposite as a new efficient catalyst for the preparation of β-amino carbonyls *via* a three-component reaction of acetophenone, aromatic aldehydes, and aniline derivatives under ultrasonic irradiation (Scheme 1).

## 2. Results and discussion

**Fig. 1:**
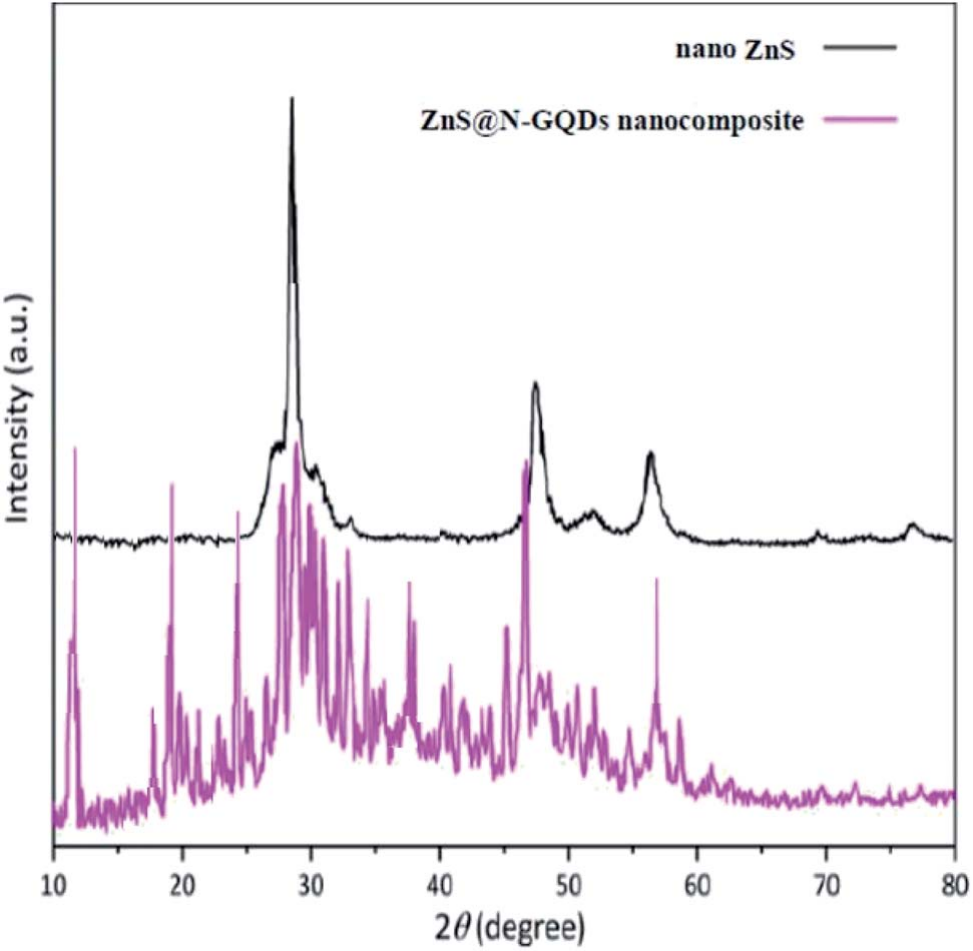
XRD pattern of the ZnS@N-GQD nanocomposite.

We prepared a ZnS@N-GQD nanocomposite *via* easy techniques. The XRD patterns of nano-ZnS and ZnS@N-GQD nanocomposite are shown in Fig. 1. The XRD patterns confirm the presence of ZnS (JCPDS no. 77-2100). XRD results have been supported by literature.^38–45^ A peak appearing at 27.3° can be indexed to the reflection of the hexagonal wurtzite phase.^39^ The peaks at 47° and 57° are attributed to nano-ZnS.^39^ XRD of the ZnS@N-GQD nanocomposite shows peaks at 11° and 24.5° that correspond to the planes of graphene structures.^42–46^ There is also a peak appearing in the range of 17–22°, corresponding the *d*-spacing of 0.37–0.52 nm.^44–46^ Maybe other peaks in the XRD pattern of ZnS@N-GQDs are related to irregular carbon structures.^47,48^

**Fig. 2:**
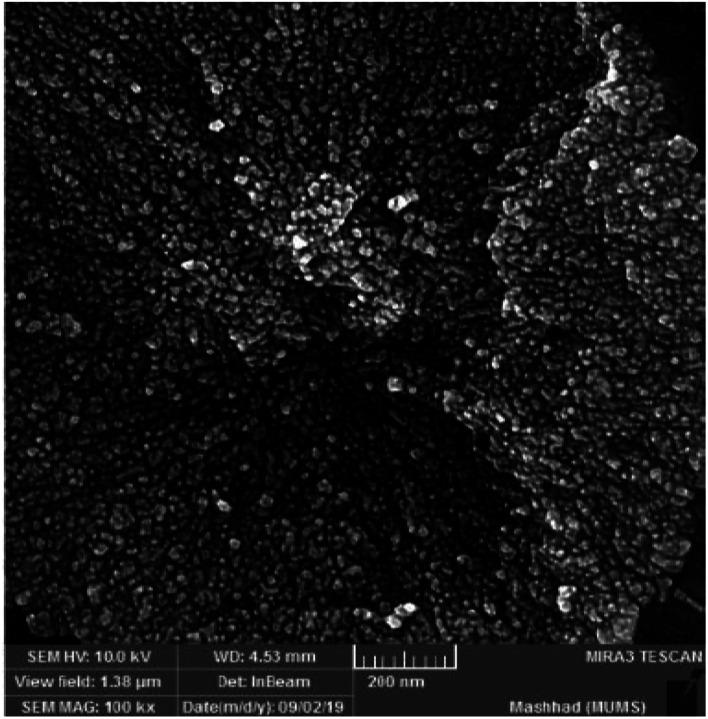
SEM image of the ZnS@N-GQD nanocomposite.

**Fig. 3:**
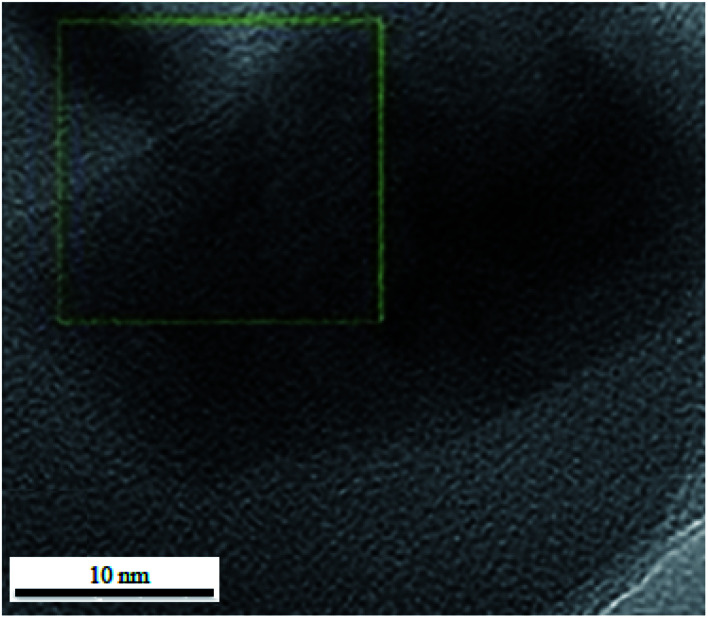
HRTEM of the ZnS@N-GQD nanocomposite.

**Fig. 4:**
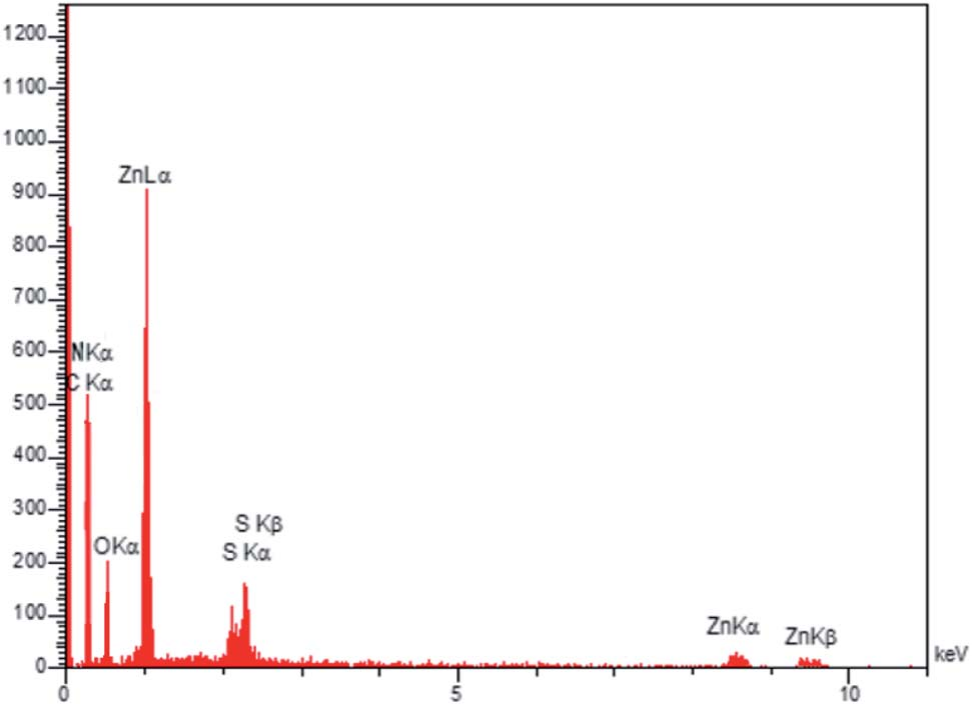
EDS image of the ZnS@N-GQD nanocomposite.

In order to investigate the morphology and particle size of the nanocomposite, the SEM (scanning electron microscopy) image of the ZnS@N-GQD nanocomposite is displayed in Fig. 2. The SEM image indicates particles with diameters in the range of nanometers. The high-resolution transmission electron microscopy (HRTEM) (Fig. 3) image of the ZnS@N-GQD nanocomposite indicated that the as-prepared nanocomposites were crystalline with lattice spacing in the range of nanometers. Note that the ZnS arrays had a rough surface that can be appropriate for the immobilization of N-GQDs. The energy-dispersive X-ray spectrum (EDS) confirmed the presence of Zn, S, O, N and C species in the structure of the nanocomposite (Fig. 4).

**Fig. 5:**
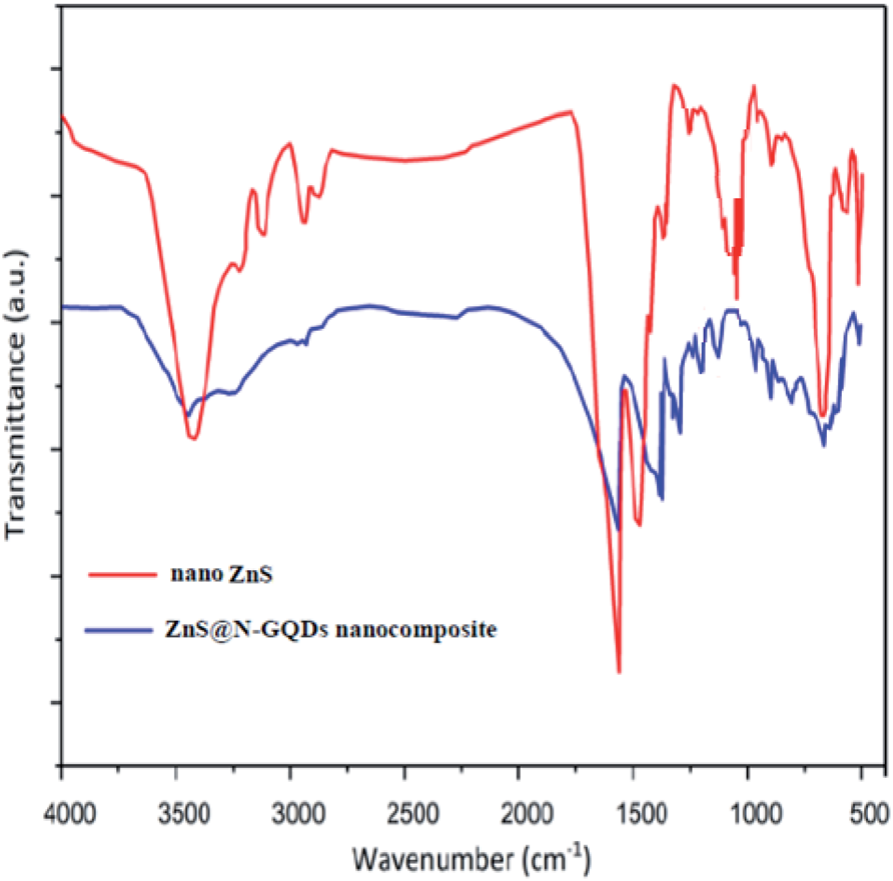
FT-IR spectra of ZnS and the ZnS@N-GQD nanocomposite.

FT-IR spectra of ZnS and the ZnS@N-GQD nanocomposite are shown in Fig. 5. The absorption peaks at 1600 and 3400 cm^−1^ are attributed to the bending and stretching vibrational absorptions of OH, respectively. The peak at 600 cm^−1^ corresponded to Zn–S. The characteristic peaks at 3434 cm^−1^ (O–H stretching vibration), 1665 cm^−1^ (C=O stretching vibration), 1103 cm^−1^ (C–O–C stretching vibration) appear in the spectrum of Fig. 5. The peak at approximately 1470–1582 cm^−1^ is attributed to C=C bonds.

The results for N_2_ adsorption–desorption containing the BET surface area (*S*_BET_) and the total pore volumes (*V*_total_) of the ZnS and ZnS@N-GQD nanocomposite are summarized in Table 1. The results presented that the BET specific surface area of ZnS was improved from 5.18 to 14.90 m^2^ g^−1^ after modification with GQDs; therefore, more active sites were introduced on the nanostructure surface.

**Table 1:** BET surface area (*S*_BET_) and the total pore volumes (*V*_total_) of the nanostructures

Materials	*S*_BET_ (m^2^ g^−1^)	*V*_total_ (cm^3^ g^−1^)
Nano-ZnS	5.18	0.08
ZnS@N-GQD nanocomposite	14.90	0.15

**Fig. 6:**
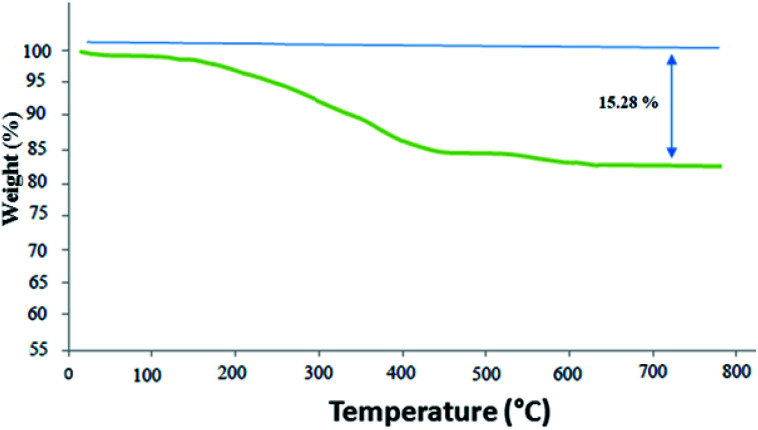
TGA of the ZnS@N-GQD nanocomposite.

Thermogravimetric analysis (TGA) considers the thermal stability of the ZnS@N-GQD nanocomposite (Fig. 6). The weight loss (2.05%) at temperatures below 200 °C is due to the removal of the physically adsorbed solvent and surface hydroxyl groups. The curve displays a weight loss about 13.23% from 200 °C to 700 °C, which is attributed to the oxidation and degradation of N-GQDs.

Initially, we investigated the three-component reaction of acetophenone, benzaldehyde, and aniline as a model reaction. The model reactions were performed using Et_3_N, NaHSO_4_, nano-ZnO, nano-ZnS and ZnS@N-GQD nanocomposite. The reactions were conducted using diverse solvents including ethanol, acetonitrile, water or dimethylformamide. The role of ultrasonic irradiation and the effects of different powers of ultrasonic irradiation (30 W, 40 W and 50 W) on the reaction were studied. The results indicated that sonication certainly affected the reaction system. It could decrease the reaction time and increase the yield of the products. When the reaction was carried out under reflux conditions (entries 6–11, Table 2), it gave lower yields of products and took longer reaction times (80 min), while the same reaction was carried out under ultrasonic irradiation to give excellent yields of products in short reaction times (10 min). The best results were gained in EtOH and we found that the reaction gave convincing results in the presence of the ZnS@N-GQD nanocomposite (8 mg) under ultrasonic irradiation (Table 2). In further studies on catalyst loading, we recognized that the yield of compound 4a remained almost the same when 9 mg of the ZnS@N-GQD nanocomposite was used. The use of the lower catalyst loading (7 mg) afforded 4a in 87% yield.

**Table 2:** Optimization of reaction conditions using different catalysts^a^

Entry	Catalyst (amount)	Solvent	Time (min)	Yield^b^ (%)
1	None	EtOH (reflux)	500	5
2	Et_3_N (5 mol%)	EtOH (reflux)	350	24
3	NaHSO_4_ (6 mol%)	EtOH (reflux)	300	55
4	Nano-ZnO (10 mg)	EtOH (reflux)	400	62
5	Nano-ZnS (10 mg)	EtOH (reflux)	250	70
6	ZnS@N-GQD nanocomposite (7 mg)	EtOH (reflux)	80	84
7	ZnS@N-GQD nanocomposite (8 mg)	EtOH (reflux)	80	90
8	ZnS@N-GQD nanocomposite (9 mg)	EtOH (reflux)	80	90
9	ZnS@N-GQD nanocomposite (8 mg)	H_2_O (reflux)	120	60
10	ZnS@N-GQD nanocomposite (8 mg)	DMF (reflux)	100	68
11	ZnS@N-GQD nanocomposite (8 mg)	CH_3_CN (reflux)	80	72
12	ZnS@N-GQD nanocomposite (8 mg)	EtOH (US: 30 W)	15	84
13	ZnS@N-GQD nanocomposite (7 mg)	EtOH (US: 40 W)	10	87
14	ZnS@N-GQD nanocomposite (8 mg)	EtOH (US: 40 W)	10	93
15	ZnS@N-GQD nanocomposite (9 mg)	EtOH (US: 40 W)	10	93
16	ZnS@N-GQD nanocomposite (8 mg)	EtOH (US: 50 W)	10	92
17	ZnS@N-GQD nanocomposite (8 mg)	H_2_O (US: 40 W)	20	62
18	ZnS@N-GQD nanocomposite (8 mg)	DMF (US: 40 W)	15	72
19	ZnS@N-GQD nanocomposite (8 mg)	CH_3_CN (US: 40 W)	10	80

a Acetophenone (1 mmol), benzaldehyde (1 mmol) and aniline (1 mmol).

b Isolated yield.

The effect of electron-withdrawing and electron-donating substituents on the aromatic ring of aldehydes and aniline on the reaction yields was investigated (Table 3). Aromatic aldehydes having nitro groups reacted at a faster rate compared with aromatic aldehydes substituted with other groups.

**Table 3:** Synthesis of β-amino carbonyls using the ZnS@N-GQD nanocomposite (8 mg) under ultrasonic irradiation^a^

Entry	Aldehyde	Aniline	Product (4a–4h)	Time (min)	Yield^b^ (%)	mp (°C) (ref.)	mp (°C) (obtained)
1	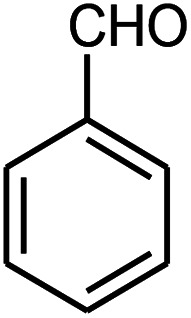	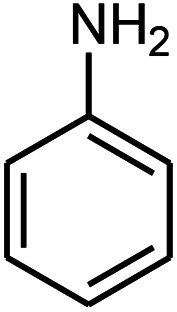	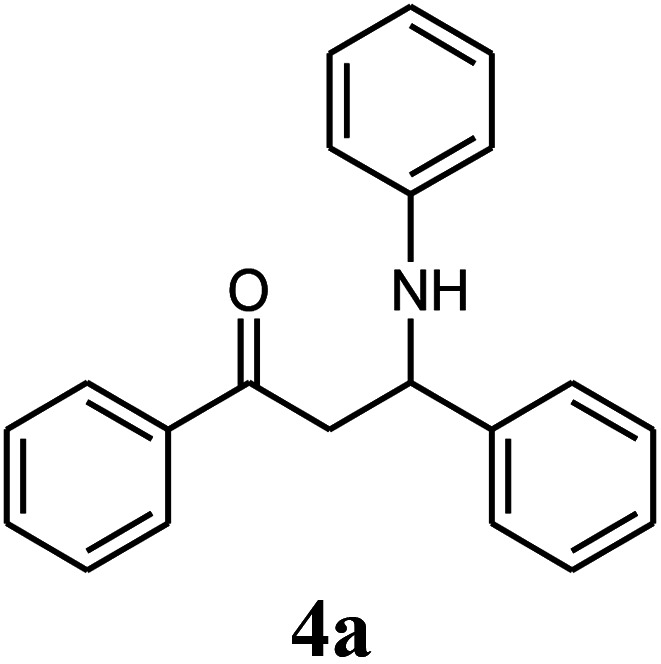	10	93	174–176 (ref. 15)	175–177
2	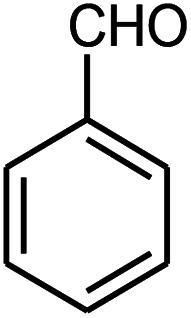	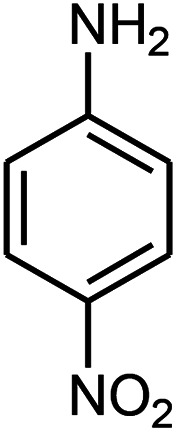	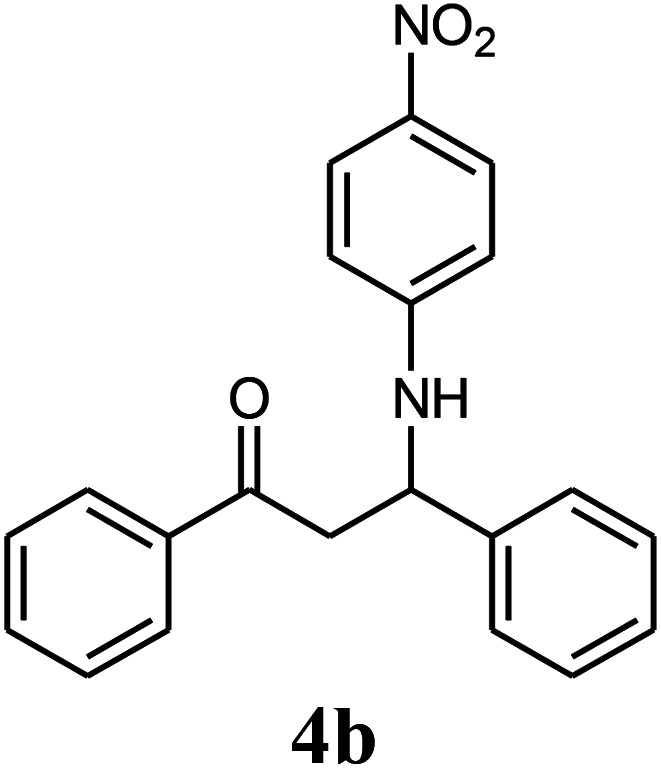	15	85	182–183 (ref. 9)	182–183
3	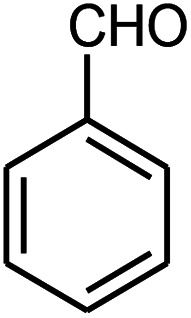	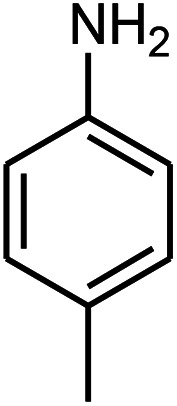	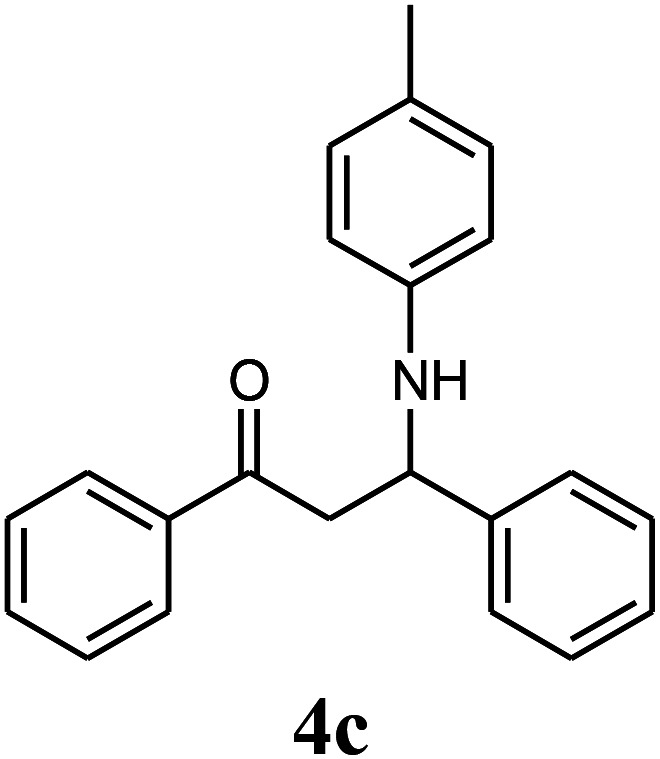	10	95	165–166 (ref. 9)	165–166
4	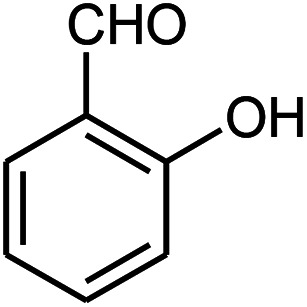	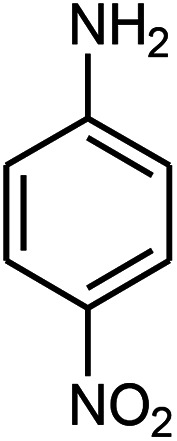	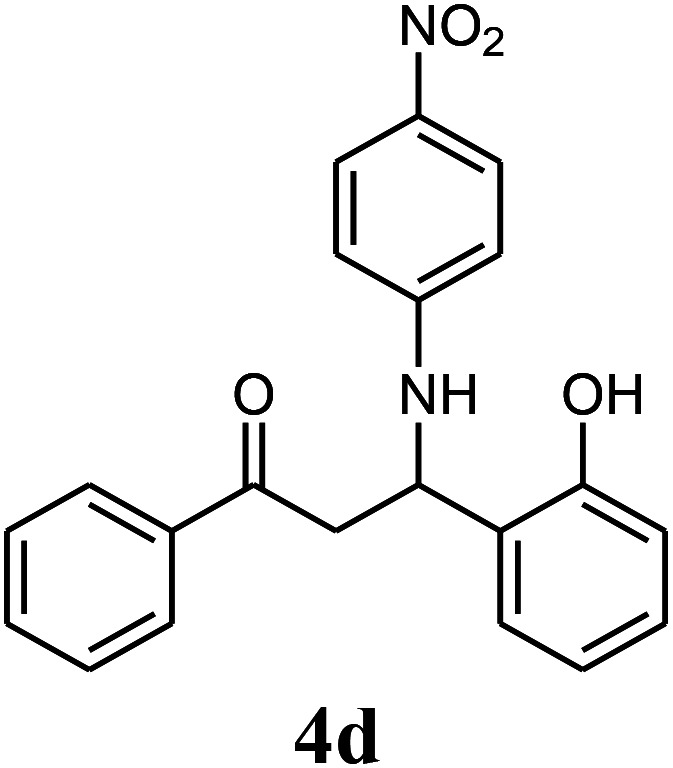	15	80		160–62
5	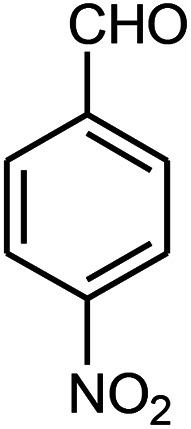	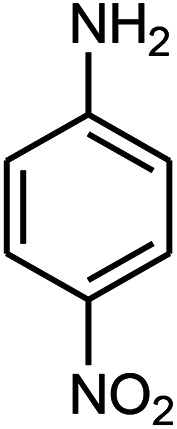	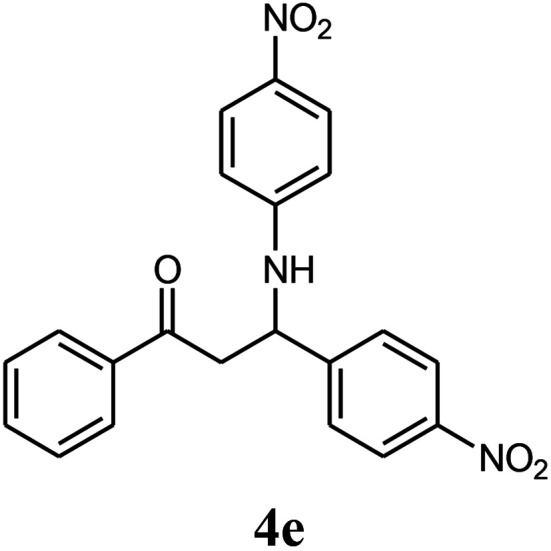	10	90		152–154
6	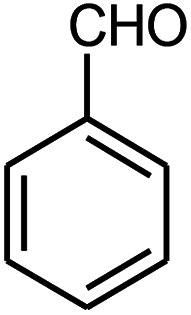	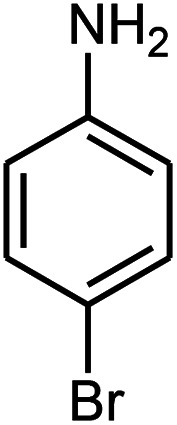	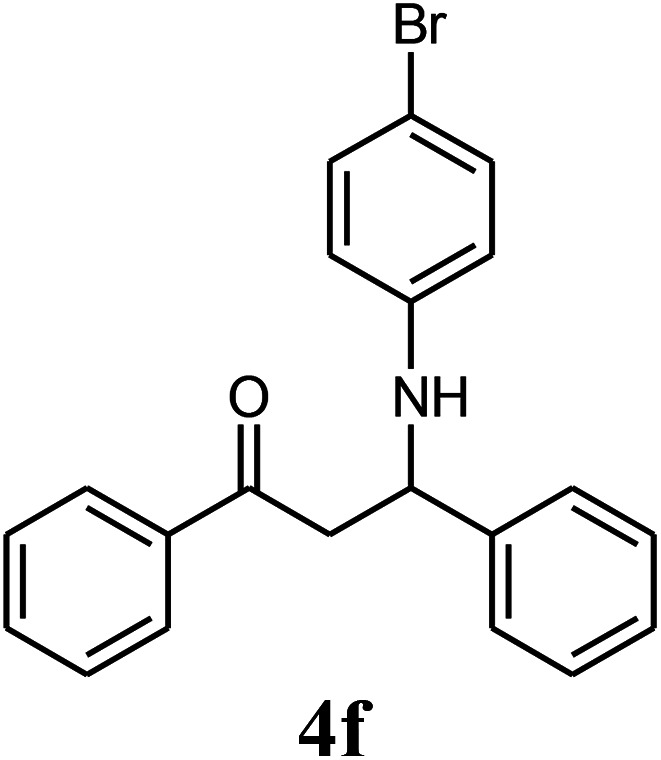	12	90	178–180 (ref. 49)	178–180
7	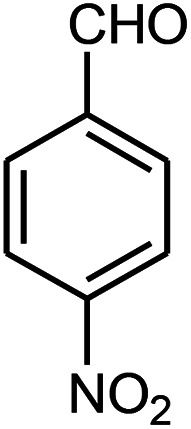	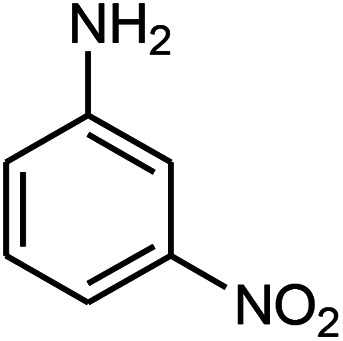	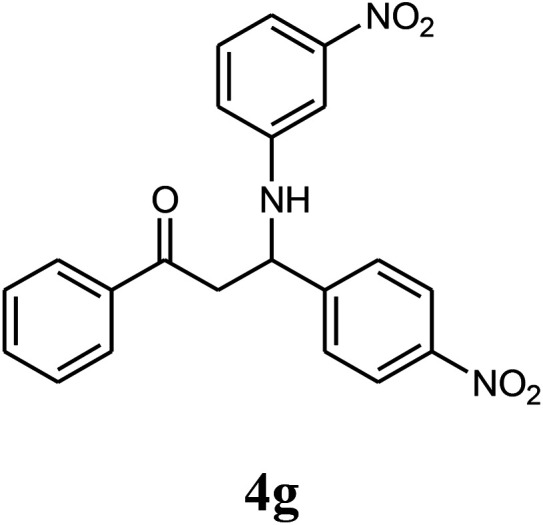	10	92		182–184
8	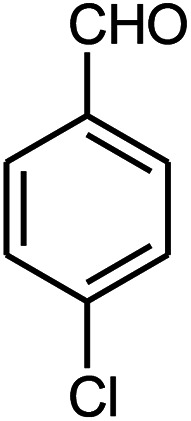	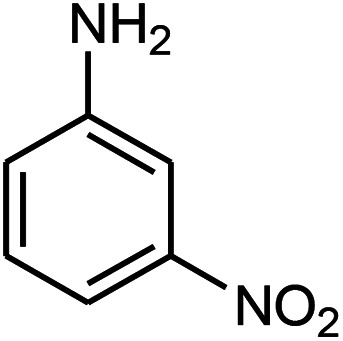	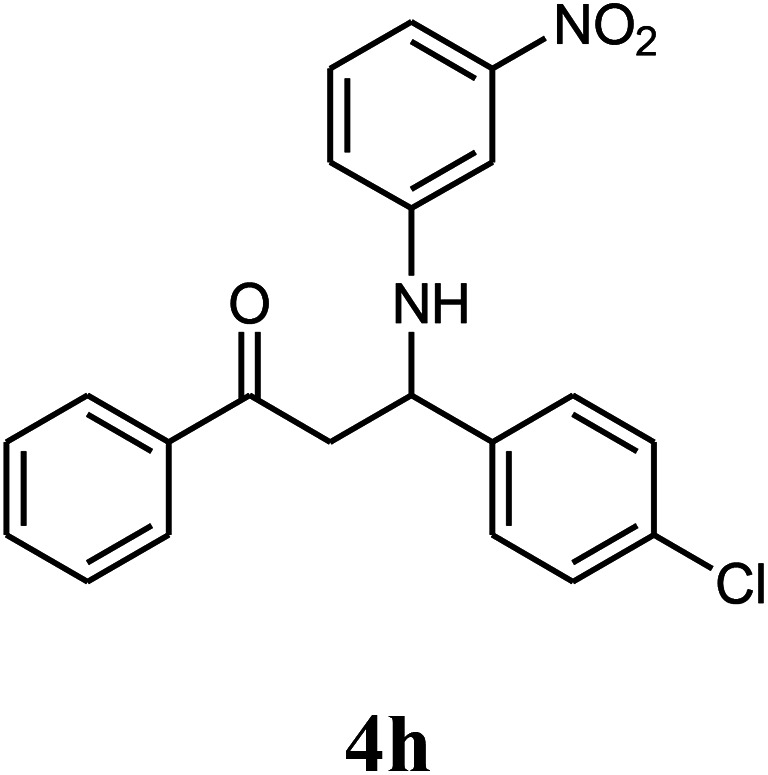	10	90		172–174

a Acetophenone (1 mmol), aromatic aldehydes (1 mmol), and aniline derivatives (1 mmol).

b Isolated yield.

**Fig. 7:**
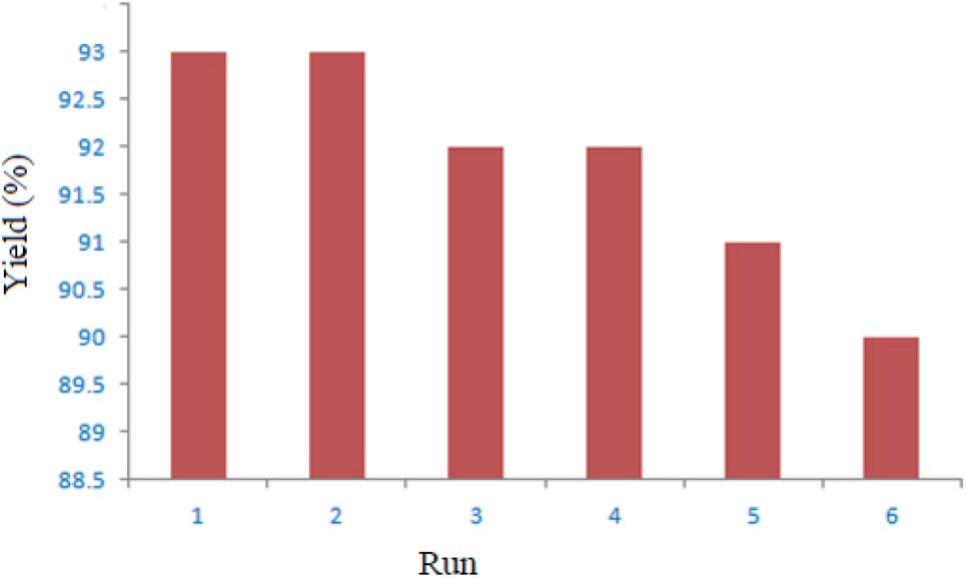
Recycling of the ZnS@N-GQD nanocomposite as the catalyst for the model reaction.

We considered the recycling of the ZnS@N-GQD nanocomposite as a catalyst for the model reaction. The results showed that the nanocomposite can be reused several times without remarkable loss of catalytic activity (yields 93 to 90%) (Fig. 7). For the recycling of the ZnS@N-GQD nanocomposite, the solution was filtered and the nanocatalyst was recovered. The recovered ZnS@N-GQD nanocomposite was rinsed four times with ethyl acetate and dried at 70 °C for 5 h.

**Scheme 2:**
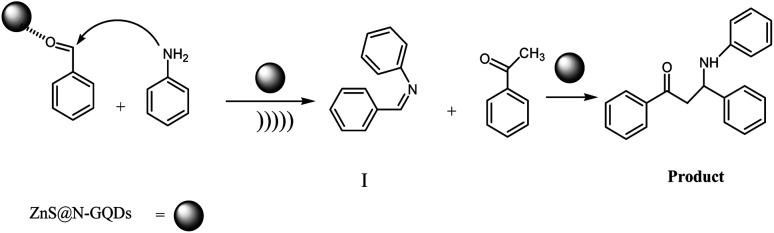
A plausible mechanism for the preparation of β-amino carbonyls using the ZnS@N-GQD nanocomposite.

A plausible mechanism for the preparation of β-amino carbonyls using the ZnS@N-GQD nanocomposite is shown in Scheme 2. First, we assumed that the reaction occurs *via* a condensation between aniline and aldehyde to form intermediate I on the active sites of the ZnS@N-GQD nanocatalyst. Then, acetophenone was added to intermediate I to afford the product. This mechanism has been supported by literature.^9,14^

To compare the efficiency of the ZnS@N-GQD nanocomposite with the reported catalysts for the synthesis of β-amino carbonyls, we have tabulated the results in Table 4. As Table 4 indicates, the ZnS@N-GQD nanocomposite is superior with respect to the reported catalysts. As expected, the increased surface area due to the small particle size increased the reactivity of the catalyst. This factor is responsible for the accessibility of the substrate molecules on the catalyst surface. Our study has some advantages in comparison with previous studies including high yield of the synthetic compound, reasonable time reaction and easy catalyst recovery.

**Table 4:** Comparison of the catalytic activity of the ZnS@N-GQD nanocomposite with other reported catalysts for the synthesis β-amino carbonyls

Entry	Catalyst (condition)	Time (min)	Yield^a^, %	Ref.
1	HClO_4_, 25 mol%, polyoxyethylene	360	90	8
2	Phenyl boronic acid, 20 mol%, CH_3_CN	640	88	9
3	Zn(OTf)_2_, 10 mol%, dichloromethane	240	88	10
4	Indium trichloride, 20 mol%, H_2_O	200	70	11
5	Ceric ammonium nitrate (CAN), 5 mol%, PEG	300	85	13
6	BiCl_3_, 5 mol%, EtOH	600	90	14
7	Sulfamic acid, 10 mol%, EtOH	300	91	15
8	ZnS@N-GQD nanocomposite, 8 mg, EtOH, (ultrasonic irradiation)	10	93	This work

a Isolated yield.

## 3. Experimental

### 3.1. Chemicals and apparatus

NMR spectra were recorded on a Bruker Avance-400 MHz spectrometer in the presence of tetramethylsilane as the internal standard. The IR spectra were recorded on an FT-IR Magna 550 apparatus using KBr plates. Melting points were determined on Electrothermal 9200 and were not corrected. Powder X-ray diffraction (XRD) was performed on a Philips diffractometer of X'pert Company with monochromatized Cu Kα radiation (*λ* = 1.5406 Å). The microscopic morphology of the nanocatalyst was visualized by SEM (MIRA3). The thermogravimetric analysis (TGA) curves are obtained by V5.1A DUPONT 2000.

### 3.2. Preparation of nano-ZnS

Nano-ZnS was prepared by simultaneously dropping 50 mL of 1 M solution of zinc sulfate and 50 mL of 1 M solution of sodium sulfide into 200 mL of distilled water containing 50 mL of 0.1 M solution of EDTA, which was vigorously stirred using a magnetic stirrer under Ar atmosphere. The precipitate was separated from the reaction mixture and was dried at room temperature.

### 3.3. Preparation of the ZnS@N-GQD nanocomposite

1 g of citric acid was dissolved into 20 mL of deionized water and stirred to form a clear solution. Then, 0.3 mL of ethylenediamine was added to the above solution and mixed to obtain a clear solution. Furthermore, 0.1 g of ZnS nanoparticles was added to the mixture. The mixture was stirred at room temperature for 5 min. The obtained solution was transferred into a 50 mL Teflon lined stainless autoclave. The sealed autoclave was heated to 180 °C for 9 h in an electric oven. Finally, as-prepared nanostructured ZnS@N-GQDs were obtained, washed several times with deionized water and ethanol, and then dried in an oven until constant weight was achieved.

### 3.4. General procedure for the preparation of β-amino carbonyls

A mixture of benzaldehyde (1 mmol), aniline (1 mmol), acetophenone (1 mmol) and 8 mg of the ZnS@N-GQD nanocomposite was stirred in 10 mL ethanol and was sonicated at 40 W power. After completion of the reaction (by TLC), the solution was filtered and the heterogeneous catalyst was recovered. Water was added, and the precipitate was collected by filtration and washed with water to give the pure product.

#### Spectra data

##### 1,3-Diphenyl-3-phenylamino-propan-1-one (4a)

White solid, mp 174–176 °C; IR (KBr, cm^−1^) 1493, 1511, 1599, (C=C, aromatic), 1670 (C=O, carbonyl), 3384 (NH, second amine); ^1^H NMR (400 MHz, CDCl_3_): 3.55 (m, 2H), 5.01 (m, 1H), 6.61 (d, 2H, *J* = 8 Hz), 6.71 (t, 1H, *J* = 7.5 Hz), 7.10 (t, 2H, *J* = 7.5 Hz), 7.26–7.32 (m, 4H), 7.44–7.46 (m, 4H), 7.53 (m, 1H), 7.89 (d, 2H, *J* = 8 Hz). ^13^C NMR (100 MHz, CDCl_3_): *δ* 46.30, 54.82, 113.85, 117.80, 126.39, 127.37, 128.21, 128.71, 128.83, 129.13, 133.44, 136.73, 142.98, 147.01, 198.28; anal. calc. for C_21_H_19_NO: C, 83.69; H, 6.35; N, 4.65; found: C, 83.62; H, 6.27; N, 4.60.

##### 3-(4-Nitrophenylamino)-1,3-diphenyl-propan-1-one (4b)

White solid, mp 182–183 °C; IR (KBr, cm^−1^) 1489, 1534, 1599, (C=C, aromatic), 1684 (C=O, carbonyl), 3367 (NH, second amine); ^1^H NMR (400 MHz, CDCl_3_): 3.55 (m, 2H), 5.10 (m, 1H), 6.52 (d, 2H, *J* = 8 Hz), 7.07 (m, 9H), 7.88 (d, 2H, *J* = 8 Hz), 7.99 (d, 2H, *J* = 8 Hz). ^13^C NMR (100 MHz, CDCl_3_): *δ* 42.42, 56.45, 112.56, 122.95, 124.22, 127.10, 128.32, 128.94, 129.13, 131.34, 133.32, 136.14, 139.40, 143.67, 187.19. Anal. calc. for C_21_H_18_N_2_O_3_: C, 72.82; H, 5.24; N, 8.09; found: C, 72.75; H, 5.26; N, 8.02.

##### 1,3-Diphenyl-3-*p*-tolylamino-propan-1-one (4c)

White solid, mp 165–166 °C; IR (KBr, cm^−1^) 1519, 1526 (C=C, aromatic), 1678 (C=O, carbonyl), 3400 (NH, second amine); ^1^H NMR (400 MHz, CDCl_3_): 2.19 (s, 3H, CH_3_), 3.53 (m, 2H), 4.97 (m, 1H), 6.38 (d, 2H, *J* = 8 Hz), 6.55 (d, 2H, *J* = 8 Hz), 6.90–7.56 (m, 9H), 7.89 (d, 2H, *J* = 8 Hz). ^13^C NMR (100 MHz, CDCl_3_): *δ* 24.20, 41.52, 55.24, 110.36, 119.68, 120.32, 121.25, 125.91, 125.82, 128.10, 128.95, 131.33, 134.27, 136.83, 145.12, 193.25. Anal. calc. for C_22_H_21_NO: C, 83.78; H, 6.71; N, 4.44; found: C, 83.68; H, 6.74; N, 4.49.

##### 3-(4-Nitrophenylamino)-3-(2-hydroxyphenyl)-1-phenylpropan-1-one (4d)

White solid, mp 160–162 °C; IR (KBr, cm^−1^) 1297, 1469, 1597 (C=C, aromatic), 1664 (C=O, carbonyl), 3364 (NH, second amine); ^1^H NMR (400 MHz, CDCl_3_): 3.56 (m, 2H), 5.41 (m, 1H), 6.51 (d, 2H, *J* = 8 Hz), 6.73–8.01 (m, 12H), 9.83 (s, 1H). ^13^C NMR (100 MHz, CDCl_3_): *δ* 43.92, 72.73, 114.45, 115.75, 121.23, 121.83, 128.32, 128.52, 128.76, 128.86, 130.95, 133.25, 136.71, 136.84, 153.74, 154.12, 199.42. Anal. calc. for C_21_H_18_N_2_O_4_: C, 69.60; H, 5.01; N, 7.73; found: C, 69.52; H, 4.94; N, 7.70.

##### 3-(4-Nitrophenylamino)-3-(4-nitrophenyl)-1-phenylpropan-1-one (4e)

White solid, mp 152–154 °C; IR (KBr, cm^−1^) 1470, 1598 (C=C, aromatic), 1657 (C=O, carbonyl), 3344 (NH, second amine); ^1^H NMR (400 MHz, CDCl_3_): 3.59 (m, 2H), 5.21 (m, 1H), 6.50 (d, 2H, *J* = 8 Hz), 7.26–8.30 (m, 12H). ^13^C NMR (100 MHz, CDCl_3_): *δ* 48.52, 72.68, 114.25, 120.84, 121.93, 127.45, 128.85, 129.35, 133.25, 136.75, 136.84, 146.52, 149.65, 153.82, 198.25. Anal. calc. for C_21_H_17_N_3_O_5_: C, 64.45; H, 4.38; N, 10.74; found: C, 64.40; H, 4.32; N, 10.71.

##### 3-(4-Bromophenylamino)-1,3-diphenylpropan-1-one (4f)

White solid, mp 178–180 °C; IR (KBr, cm^−1^) 1529, 1596 (C=C, aromatic), 1685 (C=O, carbonyl), 3372 (NH, second amine); ^1^H NMR (400 MHz, CDCl_3_): 3.48 (m, 2H), 4.95 (m, 1H), 6.46 (d, 2H, *J* = 8 Hz), 7.02 (d, 2H, *J* = 8 Hz), 7.23–7.57 (m, 9H), 7.59 (d, 2H, *J* = 7.8 Hz). ^13^C NMR (100 MHz, CDCl_3_): *δ* 48.56, 72.54, 111.42, 115.35, 126.50, 127.10, 128.52, 128.95, 130.25, 132.54, 133.82, 136.74, 143.56, 146.85, 198.54. Anal. calc. for C, 66.33; H, 4.77; N, 3.68; found: C, 66.30; H, 4.71; N, 3.62.

##### 3-(3-Nitrophenylamino)-3-(4-nitrophenyl)-1-phenylpropan-1-one (4g)

White solid, mp 182–184 °C; IR (KBr, cm^−1^) 1314, 1518 (C=C, aromatic), 1683 (C=O, carbonyl), 3414 (NH, second amine); ^1^H NMR (400 MHz, CDCl_3_): 3.56 (m, 2H), 5.16 (m, 1H), 6.81 (d, 1H, *J* = 8 Hz), 7.42–7.66 (m, 9H), 7.90 (d, 2H, *J* = 8 Hz), 8.22 (d, 2H, *J* = 8 Hz). ^13^C NMR (100 MHz, CDCl_3_): *δ* 48.32, 73.05, 107.43, 109.46, 119.82, 120.84, 127.68, 128.62, 128.37, 130.76, 133.25, 136.84, 146.48, 148.56, 149.28, 149.68, 199.14. Anal. calc. for C_21_H_17_N_3_O_5_: C, 64.45; H, 4.38; N, 10.74; found: C, 64.40; H, 4.32; N, 10.71.

##### 3-(3-Nitrophenylamino)-3-(4-chlorophenyl)-1-phenylpropan-1-one (4h)

White solid, mp 172–174 °C; IR (KBr, cm^−1^) 1483, 1525 (C=C, aromatic), 1668 (C=O, carbonyl), 3484 (NH, second amine); ^1^H NMR (400 MHz, CDCl_3_): 3.49 (m, 2H), 5.02 (m, 1H), 6.82 (d, 2H, *J* = 8 Hz), 7.19–7.61 (m, 10H), 7.89 (d, 2H, *J* = 8 Hz). ^13^C NMR (100 MHz, CDCl_3_): *δ* 49.43, 72.45, 107.43, 109.64, 119.64, 128.72, 128.83, 129.04, 129.24, 130.82, 132.58, 134.63, 136.85, 141.71, 148.83, 149.73, 198.54. Anal. calc. for C_21_H_17_ClN_2_O_3_: C, 66.23; H, 4.50; N, 7.36; found: C, 66.23; H, 4.48; Cl, 9.28; N, 7.32.

## 4. Conclusions

In conclusion, we have reported an efficient method for the synthesis of β-amino carbonyls using the ZnS@N-GQD nanocomposite as a superior catalyst under ultrasonic irradiation. The new catalyst is characterized by XRD, SEM, TEM, FT-IR spectroscopy, EDS, BET and TGA techniques. The salient features of this protocol are: great yields in concise times under sonication, retrievability of the nanocatalyst and little nanocatalyst loading.

## Conflicts of interest

There are no conflicts to declare.

## Supplementary Material
